# The genetic status and rescue measure for a geographically isolated population of Amur tigers

**DOI:** 10.1038/s41598-024-58746-9

**Published:** 2024-04-06

**Authors:** Yao Ning, Dongqi Liu, Jiayin Gu, Yifei Zhang, Nathan James Roberts, Valentin Yu Guskov, Jiale Sun, Dan Liu, Ming Gong, Jinzhe Qi, Zhijian He, Chunmei Shi, Guangshun Jiang

**Affiliations:** 1 College of Life Science, Jilin Agricultural University, 2888 Xincheng Street, Changchun, 130118 China; 2 Feline Research Center of National Forestry and Grassland Administration, College of Wildlife and Protected Area, Northeast Forestry University, Harbin, China; 3 Federal Scientific Center of the East Asia Terrestrial Biodiversity, Far Eastern Branch of Russian Academy of Sciences (FSCEATB FEB RAS), Vladivostok, Russian Federation; 4 Siberian Tiger Park, Harbin, 150028 Heilongjiang China; 5 College of Mathematics and Computer Science, Zhejiang Agriculture and Forestry University, Hangzhou, China

**Keywords:** Amur tiger, Genetic diversity, Effective population size, Inbreeding, Population viability, Ecology, Genetics, Zoology

## Abstract

The Amur tiger is currently confronted with challenges of anthropogenic development, leading to its population becoming fragmented into two geographically isolated groups: smaller and larger ones. Small and isolated populations frequently face a greater extinction risk, yet the small tiger population’s genetic status and survival potential have not been assessed. Here, a total of 210 samples of suspected Amur tiger feces were collected from this small population, and the genetic background and population survival potentials were assessed by using 14 microsatellite loci. Our results demonstrated that the mean number of alleles in all loci was 3.7 and expected heterozygosity was 0.6, indicating a comparatively lower level of population genetic diversity compared to previously reported studies on other subspecies. The genetic estimates of effective population size (*Ne*) and the *Ne/N* ratio were merely 7.6 and 0.152, respectively, representing lower values in comparison to the Amur tiger population in Sikhote-Alin (the larger group). However, multiple methods have indicated the possibility of genetic divergence within our isolated population under study. Meanwhile, the maximum kinship recorded was 0.441, and the mean inbreeding coefficient stood at 0.0868, both of which are higher than those observed in other endangered species, such as the African lion and the grey wolf. Additionally, we have identified a significant risk of future extinction if the lethal equivalents were to reach 6.26, which is higher than that of other large carnivores. Further, our simulation results indicated that an increase in the number of breeding females would enhance the prospects of this population. In summary, our findings provide a critical theoretical basis for further bailout strategies concerning Amur tigers.

## Introduction

Biodiversity sustains the functioning of ecosystems; however, under the effect of climate change, economic development activities, and human land use, almost 28 percent of the 150,388 species surveyed in the most recent update of the International Union for the Conservation of Nature’s (IUCN) Red List have been identified as threatened with extinction, and species conservation has become a critical issue facing mankind^1–4^. For wild animals, when heavily plagued by habitat loss or fragmentation, prey shortage, viruses, inbreeding depression, small population size, and other factors, it may indicate that the species is in an extinction vortex^5–8^. Previous studies have employed models to discover why small and isolated populations have an increased risk of extinction due to the interplay between genetics and demography^9^. Assessing the allelic richness of species and populations is widely employed to quantify genetic diversity, thereby evaluating fitness and adaptability in response to environmental changes^10–12^.

Genetic diversity, one of the three aspects of biodiversity (including genetic diversity, species diversity, and ecosystem diversity), is regarded as a parameter that effectively reflects the variability of the demographic and historical population processes^13,14^. Defining the amount and distribution of genetic variation within and among populations by using genetic structure is a crucial research direction in the fields of genetics and bioinformatics^15,16^. The effective population size, a core evolutionary parameter, exerts an influence on the rate of genetic diversity loss as large populations could store greater genetic variation while governing the mating selection space, affecting the probability of inbreeding within populations^17–19^. These parameters are the classic components of population genetics, measured across samples of multiple populations of the same species, and serve as fundamental indicators for evaluating genetic status^20–24^.

When the population is small and isolated, a common phenomenon is that the offspring of individuals mating with close relatives are likely to inherit two copies of the same recessive deleterious allele, which are present in the population but rarely expressed, thus suffering the consequences of harmful gene expression and production of deleterious traits, defined as inbreeding depression^25^. These pernicious qualities of inbreeding depression typically decrease individual fitness, interfere with the long-term sustainable survival of the population, and even drive the population to impasse^26–29^. A real historical case is the Isle Royale gray wolf (*Canis lupus*) population, which, after 70 years of isolation, changed from an original population of 10–50 individuals to only three individuals in 2015 due to inbreeding depression^27,30,31^. Although the pack social structure of wolves may result in a higher likelihood of inbreeding compared to the solitary lifestyle of tigers, now this similar situation of the small and isolated population once more occurs in the population of Amur tiger (*Panthera tigris altaica*). Undoubtedly, there is an urgent need to investigate the degree of inbreeding within the population in order to scientifically implement rescue measures and alleviate the situation^32,33^.

The Amur tiger is the most northerly subspecies of tiger and possesses the largest home range among tiger subspecies^34,35^. More than 50 individuals have been recorded in China through comprehensive surveys, and they are distributed in four major forested landscapes (Lesser Khingan Mountains, Wanda Mountains, Zhangguangcailing, Laoyeling)^36^. At the same time, 750 individuals in Russia are divided into two distinct subpopulations; the smaller population is located in Southwest Primorye, geographically adjacent to the Laoyeling landscape, which constitutes the major tiger range in China^32,37^. The gene flow between this isolated population and the larger population in Sikhote-Alin Mountains was obstructed by the exacerbation of urbanization, which included the construction of roads, agricultural lands, and small business complexes^33,38^. Adequate surveys and protection of this small isolated cross-border population, which is from Laoyeling and Southwest Primorye, will help to devise the implementation of conservation strategies that will aid the expansion of the large tiger population from Russia to China to address the environmental carrying capacity burden of the concentrated tiger population in Sikhote-Alin Mountains while ensuring the steady increase of the tiger population in China and furthering the spread to the other three forested landscapes. Ultimately, this is expected to lead to achieving the long-term goal of sustainable survival of the wild Amur tiger and ecosystem stability. However, there are still research gaps in investigating the current genetic variation in Laoyeling and Southwest Primorye, measuring the degree of inbreeding, and detecting the effect of inbreeding depression on future population viability.

For this research, we used genetic samples of Amur tiger in the small and isolated cross-border populations in China and Russia to test three specific hypotheses: (1) genetic diversity of this population has reached a moderate level based on the various conservation strategies targeting the Amur tiger, which have been implemented over time; (2) there is a paucity of individuals with close kinship due to the increasing trend and expansion of mating territories among Amur tigers; 3) next, the most effective approach to guarantee the sustainable persistence of this population would be to release a specific number of breeding females into their natural habitat. Our study analyzed the intrinsic genetic status of the Amur tiger population based on microsatellite data, and the results of this study provide a basis for further implementation of the tiger population rescue program.

## Materials and methods

### Study area and sampling methodology

**Figure 1:**
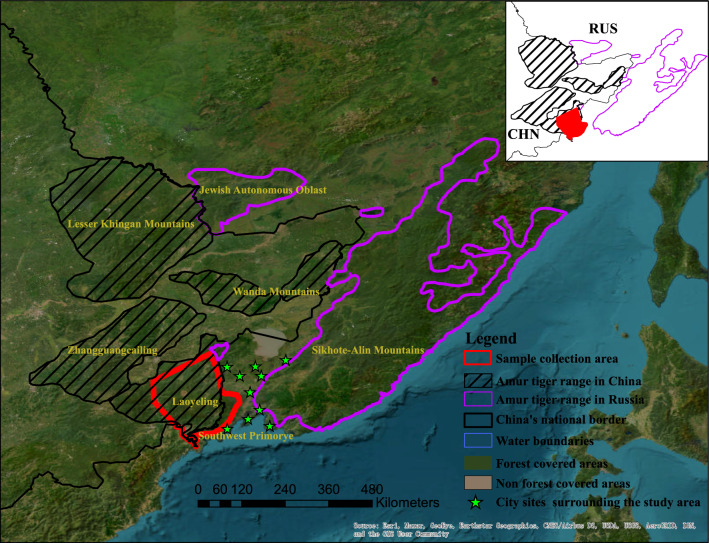
The geographical distribution map of the study is presented, with red outlines demarcating the collection areas for fecal samples in the Laoyeling Mountain of China and Southwest Primorye of Russia. Green stars indicate urban locations that impede individual dispersal to large populations.

The research area in China was limited to 18,029 km^2^ in the Laoyeling landscape, which covers the spatial distribution of all tigers recorded in Northeast China Tiger and Leopard National Park (NTLNP) and almost the entire extent (Fig. 1). The climate can be characterized as a continental humid monsoonal. The primary prey in the study area are wild boar (*Sus scrofa*) and roe deer (*Capreolus pygargus*), with the highest abundance close to 5.03 individuals/km^2^ and 12.87 individuals/km^2^, respectively^39^. Based on three different approaches of transect surveys, individual tracking, and sample delivery from local forestry bureaus or reserves, we conducted comprehensive sample collection in this area (fecal samples), and the data from the Land of Leopard National Park in Southwest Primorye, Russia, were obtained as in Ning et al*.*^40^.

### Genetic data acquisition

DNA from fecal samples was extracted by QIAamp Fast DNA Stool Mini Kit, and the operation steps followed the kit instructions with minor modifications such that we only pipetted 100 µl Buffer ATE onto the QIAamp membrane and incubated for 5 min at room temperature in the final step of the procedure. All samples were first identified with species-specific primers Pta-CbF/Pta-CbR to identify the Amur tiger samples, and the identified samples were subjected to multiplex PCR assay with a total of four panel 18 microsatellite loci (FCA5, FCA32, FCA43, FCA44, FCA69, FCA77, FCA90, FCA94, FCA105, FCA161, FCA176, FCA211, FCA220, FCA290, FCA293, FCA304, FCA310, FCA391)^41,42^. The reaction system and PCR amplification conditions were consistent with previous studies, and GENEMAPPER v4.0 was used to detect different allele sizes^40^. We conducted genotyping four times, followed by the determination of the genotype with a standard peak map. Simultaneously, the consensus genotype was determined, requiring a minimum of three occurrences for homozygotes and two occurrences for heterozygotes^43^. Using Excel Microsatellite Toolkit v3.1.1, individual identification was carried out based on the criteria that all alleles were identical or had only one mismatch, and the number of alleles (*Na*) and observed heterozygosity (*H*_*O*_), expected heterozygosity (*H*_*E*_), and the polymorphism information content (*PIC*) at each site were also calculated. The F_is_, unbiased estimation of Hardy–Weinberg equilibrium and the exact tests of genotypic disequilibrium with 10,000 dememorization, 20 batches, and 5,000 iterations per batch were performed using GENEPOP (version 4.2.1)^44^.

### Data analyses

To explore the genetic population structure of the Laoyeling landscape and Southwest Primorye, which are geographically adjacent but cross the national boundaries, we applied three different approaches. First, we used STRUCTURE software, a key tool for Bayesian-based clustering analysis, to detect the likely number of ancestral populations that fuelled into the current population^45^.We assume that the allele frequencies were correlated under the admixture model, the length of the Burnin period and the number of MCMCs Reps after Burnin was set to 100,000 and 1,000,000, respectively, while the K values of clusters ranged from 1 to 10, and each K value was repeated five times. According to the method of Evanno et al., the best fitting K value was selected in STRUCTURE HARVESTER, and we set 0.9 as the threshold for multi-locus genotypes to be classified into clusters^46^. Second, we calculated the genetic distance matrix between individuals using *D*_*sw*_, which was suitable for loci with high mutation rates, and built a phylogenetic tree using POPULATION and visualized phylogenetic relationships using TreeView v1.6.6^47,48^. Thirdly, Discriminant analysis of principal components (DAPC) was calculated using the adegenet package in R to identify and describe genetic clusters, and for drawing the scatterplots of DAPC, we divided the natural tiger individuals into three groups according to the result of STRUCTURE^49,50^.

In addition, we calculated the effective population size (*N*_*e*_) of this endangered population using the most common method of linkage disequilibrium (LD), which is considered to be more accurate than other methods, especially for small effective population sizes^51,52^. We used NeEstimator (version 2.1) to calculate the *N*_*e*_ value while setting the pairing pattern to random mating and *P*crit = 0.02 because the number of individuals sampled for genetic analysis was more than 25^52–54^.

The kinship between individuals and the inbreeding coefficient was evaluated using COANCESTRY (version 1.0.1.7) following the software user guide. First of all, we selected four common kinships, including full-siblings, half-siblings, cousins, and non-relationships, to conduct 2000 simulations of each relationship based on the observed microsatellite allele frequency. The method of kinship calculation was chosen by comparing it with the actual kinship value and selecting the method with the minimum variation value. Moreover, the inbreeding coefficient was calculated using the triadic likelihood estimator (TrioML) method and followed the widely used criterion that “high inbreeding individuals” are those with f ≥ 0.25 and “moderate inbreeding individuals” are defined as those with 0.125 ≤ f < 0.25^55,56^.

To explore whether this small and isolated population was at risk of future extinction based on the current inbreeding situation, we used Vortex (version 10.5.5) to evaluate the population viability over the next 100 years with 1000 iterations^57^. The depression severity of first-year survival resulting from inbreeding was quantified by the coefficient of lethal equivalents, in which one lethal equivalent can be interpreted as one death caused by a group of deleterious alleles if they are made homozygous^58,59^. Previous studies have proven that this value plays a decisive role in the sustainable survival of the population^60^. A single-factor sensitivity test was also used to analyze the effects of different lethal equivalent values of 0, 3.14, 6.26, and 12.26 on future survival probability to determine the critical value of inbreeding depression to maintain the sustainable survival of the population. Finally, we manipulated the ratio of reproductive females and recessive lethal alleles to investigate the rescue strategy for mitigating population extinction in this small population at each lethal equivalent using Vortex. The parameters set for the distribution of broods per year, the number of progenies, and the sex ratio at birth were based on the last five years of recorded cub births at the Siberian Tiger Park, Harbin, China, which has raised over 400 captive Amur tigers. The upper limit of the population size we simulated was derived from the published supportable density of Amur tigers (0.77 individuals/100km^2^) multiplied by the area of suitable habitat (21,254km^2^)^39^. The remaining parameters were based on default software settings, published literature, or long-term experience of studying the species (more than 20 years); refer to Table S1 for parameter details.

## Results

### Genetic diversity

A total of 210 non-invasive genetic samples were collected from this isolated population, out of which 190 samples were identified as belonging to the Amur tiger species through amplification with species-specific primers. The detailed sample sources can be found in Table S2. Finally, microsatellite data was successfully obtained from 48 samples at 14 loci, as four loci (FCA5, FCA77, FCA211, and FCA391) were removed due to amplification failure or low polymorphism (raw data). After analyzing 14 microsatellite loci, a total of 30 Amur tiger individuals were identified. We found that 21 individuals were geographically sampled from the Laoyeling landscape, and 9 individuals were sampled from Russia. The F_is_ values ranged from -0.1937 to 0.3915, with positive values indicating an excess of homozygotes and departure from Hardy–Weinberg equilibrium (Table S3)^61^. Consistent with the above results, when the data were examined for 30 individuals as a whole, FCA220 and FCA310 deviated from Hardy–Weinberg, and five sets of pairs were in linkage disequilibrium (Table S3 and S4). However, when all individuals were divided into three groups based on the STRUCTURE results, all results were unbiased, implying the presence of the Wahlund effect. The maximum *Na* in the locus was 5, and the mean value of alleles in all loci was 3.7.* H*_*E*_ of the locus ranged from 0.4 to 0.8, with a mean value of 0.6, which is identical to the mean observed (*Ho*) value (Table 1 and Table S5). The minimum and maximum values of *PIC* were 0.294 and 0.699, respectively, and the mean value was 0.519, which means that most loci have high informative values (Table S5).

**Table 1:** Comparative analysis of genetic diversity in the wild Amur tiger population between the present study and previous research.

Sampling location	Sample type	Sample size	Total number of individuals	Number of microsatellite loci	Mean *Na*	Mean *H*_*E*_	Mean *Ho*	Reference
Laoyeling landscape, Southwest Primorye (China and Russia)	feces	210	30	14	3.7	0.6	0.6	This study
SikhoteAlin Mountains, southwest Primorye (Russia)	feces, hair, blood	274	63	9	SikhoteAlin Mountains = 3.33; Southwest Primorye = 3.56	SikhoteAlin Mountains = 0.52; Southwest Primorye = 0.62	SikhoteAlin Mountains = 0.57; Southwest Primorye = 0.61	Sorokin et al*.* (2016)
Hunchun Nature reserve (China)	feces, hair	56	7	9	2.56	0.369	0.455	Wang et al*.* (2016)
southwest Primorye (Russia)	feces, hair, saliva	286	12	10	3.2	0.58	0.59	Sugimoto et al*.* (2012)
SikhoteAlin Mountains (Russia)	blood, tissue	15	15	18	2.92	0.54	0.46	Alasaad et al*.* (2011)
Hunchun Nature reserve (China)	feces	11	5	11	2.55	0.438	0.603	Caragiulo et al*.* (2015)
Hunchun reserve, Laoyeling reserve, Huangnihe reserve (China)	feces	167	11	10	2.6	0.42	0.49	Dou et al*.* (2016)

Mean *Na* = mean number of alleles per locus; Mean *Ho* = mean observed heterozygosity; Mean *H*_*E*_ = mean expected heterozygosity.

### Within-population genetic structure

**Figure 2:**
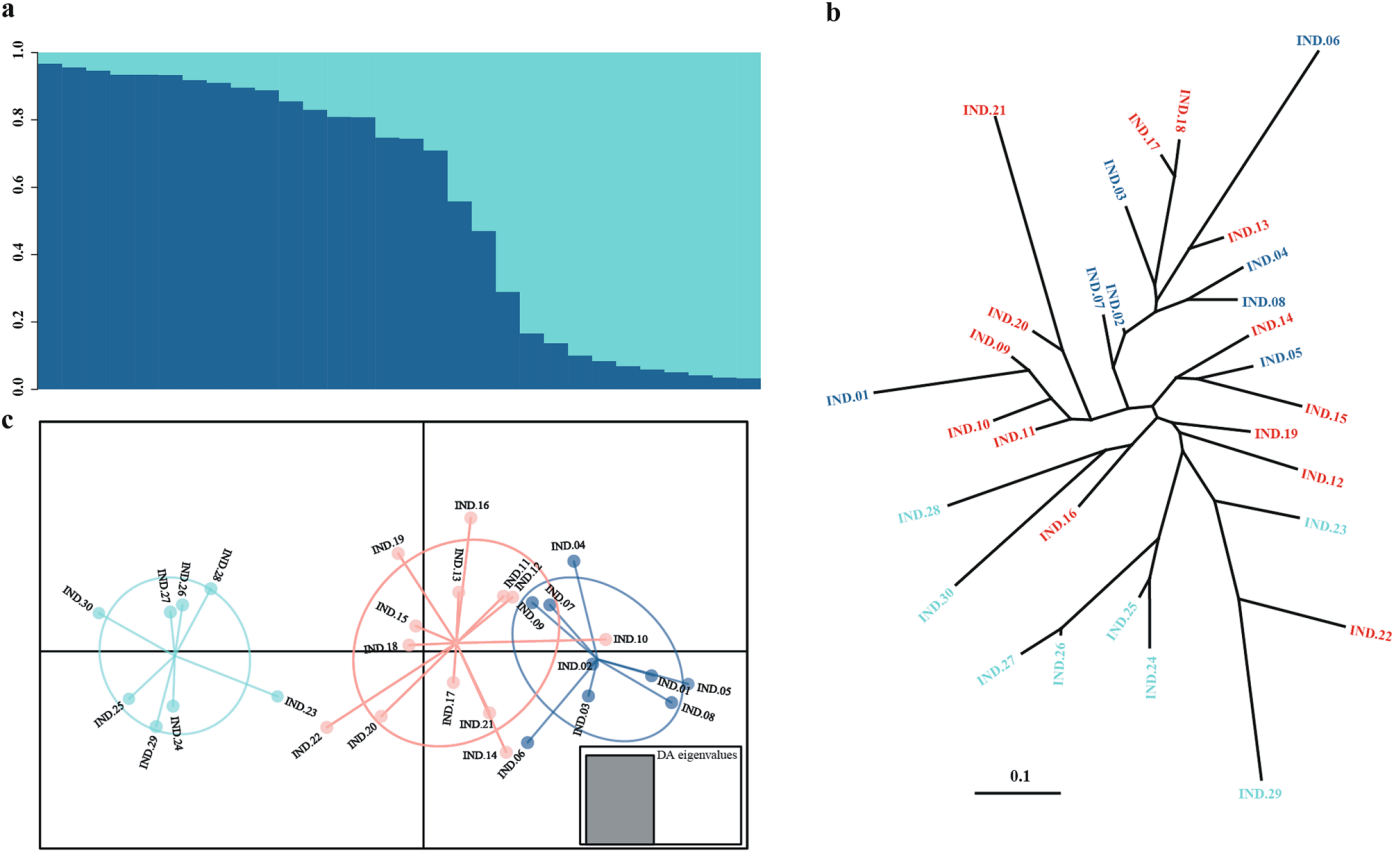
Analysis of the genetic structure of the population. A Bayesian clustering approach was applied to investigate the population's genetic structure using STRUCTURE software. According to Evanno's method, the optimal value of K was determined as 2. Each individual was represented by a vertical bar and labeled from left to right in the order of IND.1 to IND.30. (**a**) Phylogenetic relationships among 30 tiger individuals were inferred using the *Dsw* method based on data from 14 microsatellite loci. (**b**) Scatterplot of DAPC to presume the genetic architecture. (**c**) The numbers and colors are assigned to the panels. (**b**) and (**c**) were consistent with the STRUCTURE analysis. The color blue represents individuals belonging to cluster 1, green represents individuals belonging to cluster 2, and red indicates potential admixture individuals.

Bayesian clustering analysis using STRUCTURE indicated that the most likely value of K was 2, as Delta K had a peak at a horizontal coordinate of 2 (Fig. S1). When the threshold of assignment probability was set to 0.9, 53.33% (16/30) of individuals collected in the sample were assigned to one of the two clusters, and the rest were identified as potentially admixed individuals with partial contributions from both clusters (Fig. 2a and Table S6). The average genetic distance (*D*_*sw*_) was 0.494 among 30 individuals (Table S7). The neighbor-joining phylogenetic tree formed three distinct groups: group 1 consisted of IND.05, IND.14, and IND.15; group 2 comprised IND.12,16,19 and 22–30; and the remaining individuals were classified into group 3. The observed clustering pattern of individuals was generally consistent with STRUCTURE results, with individuals in cluster 1 being in groups 1 and 3, while cluster 2 was distributed in group 2, and genetically admixed individuals were distributed among the groups (Fig. 2b). In DAPC, the number of PCs retained was four according to the value of highest mean success and the lowest MSE. The DAPC analysis revealed a clear demarcation between cluster 1 and cluster 2, with no overlap observed. Additionally, the genetically mixed individuals were distributed across both clusters, exhibiting some degree of overlap, specifically with cluster 1. Importantly, these findings align well with the results obtained from the STRUCTURE analysis (Fig. 2c). Thus, all three methods based on allele frequencies (STRUCTURE) and pairwise genetic distances (Phylogenetic relationship and DAPC) obtained consistent results.

### ***Estimating contemporary N***_***e***_

Genetic estimates of *N*_*e*_ derived from the LD single-sample method based on molecular co-ancestry among 30 individuals was 7.6 (95% interval: 5.1–10.8 individuals) when the criterion for excluding rare alleles was set to 0.02 (Table S8). Based on camera trap data from our previous study, we identified 50 individual Amur tigers in Laoyeling Landscape of China. Using this number as the census size value, the *N*_*e*_/*N* ratio was 0.152, which falls within the range of the theoretical and empirical estimates for most populations (0.1–0.5)^36^.

### Analysis of the degree of inbreeding

We applied the TrioML method with the minor variation compared with the actual value to estimate the genetic relationship between individuals of this isolated population. The maximum kinship was 0.441, and the mean value was 0.134. According to the inbreeding coefficient results, 9 out of 30 individuals can be described as belonging to medium or high inbreeding classes, including six in China and three in Russia, with inbreeding coefficients 0.151, 0.165, 0.168, 0.219, 0.274, and 0.356 in China, and 0.202, 0.301, and 0.308 in Russia. The mean inbreeding coefficient determined by TrioML was 0.0868 (Table S9).

### Testing the impact of inbreeding depression

**Figure 3:**
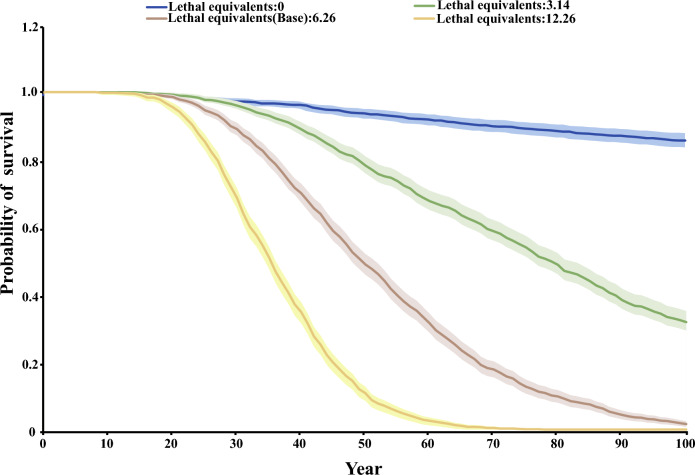
Line plots depict the trajectory of population survival probability over the next 100 years. Note: Different colored lines represent the probabilities of population survival in diverse lethal equivalent scenarios, and the lightly shaded area represents the 95% confidence interval for these trajectories.

**Figure 4:**
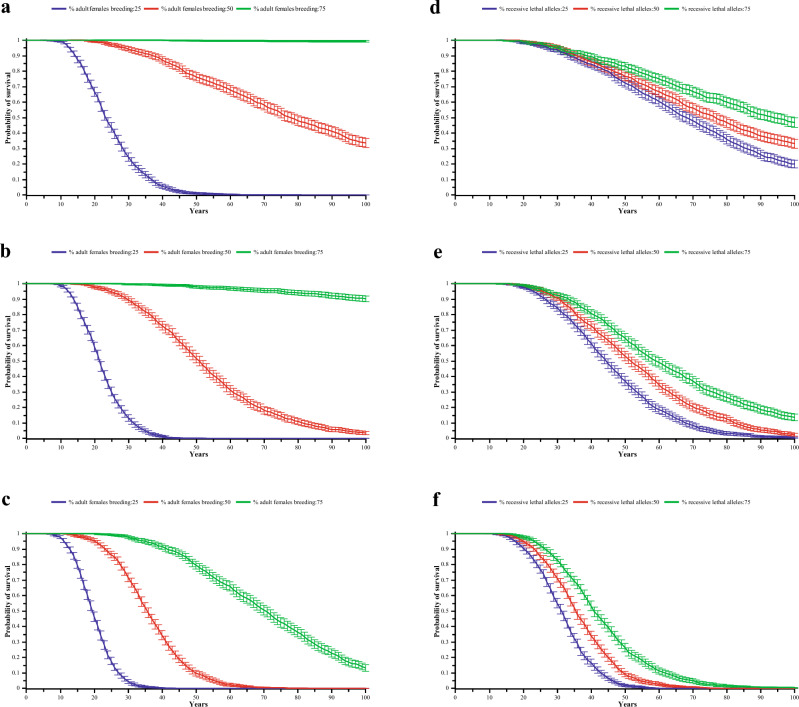
Line graph depicts the trajectory of the survival probability for this small and isolated population over the next century. (**a**,**b**,**c**) represent the survival status of this small Amur tiger population when lethal equivalent values are 3.14, 6.26, and 12.26, respectively, and different colored lines indicate our simulations for adult breeding female ratios of 25%, 50%, and 75%. The population survival possibilities simulated with lethal equivalents of 3.14, 6.26, and 12.26 were represented by (**d**,**e**,**f**), respectively. The different colors represent varying proportions of diverse recessive lethal alleles, and the 95% confidence interval is represented by error bars.

Based on the above results, the parameters for inbreeding and kinship of the starting populations in Vortex were set to 0.0868. The future trajectory of population survival probability was estimated assuming a lethal equivalent value of 6.26, and the results demonstrate an increase in population size over the initial 11-year period, then a sharp population decline in the later years. The 95% confidence interval of mean population size was larger in the next 5–60 years than in the future 61–100 years, and the probability of the population becoming extinct over a 100-year period was 98% (Fig. S2). We also performed sensitivity analyses with different lethal equivalents to measure the effect of inbreeding depression on the probability of survival and population size in this small and isolated population. The results demonstrate an inverse relationship between the value of lethal equivalents and the probability of future survival (Fig. 3). When the population lethal equivalent was 12.26, the probability of survival of the population decreased to 0 within the next 78 years. If there was no inbreeding depression in the population, the survival probability of the population in the next 100 years was 86.5%, and the number of individuals in the population was 94 in the 100th year (Fig. 3). When the lethal equivalent value is 0, this small Amur tiger population almost not face extinction risk in the future (Fig. 3). When the lethal equivalent value is 0, this small Amur tiger population almost not face evaded extinction risk in the future (Fig. 3). We chose 3.14, 6.26, and 12.26 in three lethal equivalent values to explore the optimal approach for rescuing this small population by manipulating the number of breeding females or adjusting the ratio of harmful recessive alleles. The results indicate that when the proportion of reproductive individuals reaches 75%, there is a likelihood of survival over 90% in the 100th year with lethal equivalent values of 3.14 or 6.26 (Fig. 4a,b). However, if the ratio of breeding female individuals drops to 25%, the population may become extinct within 50 years (Fig. 4a,b,c). Additionally, when the proportion of deleterious genes in the population is reduced from 75 to 25%, the prospective survival probabilities increase modestly, ranging from19.9% to 46.7%, from 0.4% to13.5%, and from 0 to 0.3% for lethal equivalents values of 3.14, 6.26, and 12.26, respectively (Fig. 4d,e,f).

## Discussion

As the top predator in the food web, the flagship species for ecological conservation, and with 13 tiger range countries reaching a consensus at the World Tiger Forum in 2010 to double the number of tigers in the wild by 2022, tiger-related developments have become even more of a popular concern. Russia currently has the world’s largest population of wild Amur tigers, 95% of which are found in the Sikhote-Alin mountain range, and a smaller percentage are found in the Southwestern Primorsky region of Russia, which is connected to the core distribution area of Amur tigers in China^32^. Molecular genetic analysis of individuals from each region in Russia in recent years revealed that rapid urbanization has led to difficulties in genetic flow between the two populations in Russia^33^. What’s more, small and isolated populations can present a high risk of continued population decline or even extinction due to their own factors or long-term disturbance by external factors of stress. Therefore, our study focuses on this small and endangered population and provides an in-depth and systematic investigation of its genetic background and assessment of future status.

### Level of genetic diversity

Genetic diversity of the population was measured using two parameters, *H*_*E*_ and *Na*, which minimize the effects of sampling bias and use a different number of markers and are applied in numerous studies^62^. The mean values of *H*_*E*_ and *Na* for the 14 loci we calculated in this population were 0.6 and 3.7, respectively, which are somewhat in accord with the previous research of 0.58–0.62 for *H*_*E*_ and 3.2–3.6 for *Na* calculated for individuals from Southwest Primorye (Table 1)^33,63^. However, our calculated values were considerably higher than those calculated from another Chinese study, which may be due to the reason that the present study is calculated using 30 individuals collected both in China and Russia rather than China-only (Table 1)^62,64,65^. While these values are a little higher than the large Sikhote-Alin population (Table 1), they are comparatively lower than those of the Bengal tiger (*P. t. tigris*) and Indochinese tigers (*P. t. corbetti*), consistent with a common perception that the genetic variation between the large and small populations of wild Amur tigers in Russia is roughly similar and lower genetic variation compared to other subspecies^32,33,66–70^. Based on our result, the genetic variation of this small and isolated population is at a low level, which can be explained by two possible reasons: one, a recent genetic bottleneck in Amur tiger was identified by applying 11 loci in 15 individuals, and the results coincide with a severe demographic decline of the Amur tiger population in the 1940s^66^; second, a historical bottleneck possibly related to the founder effect of the Amur tiger's Far Eastern range during post-ice age colonization^32^.

In summary, as highlighted by Wilting et al., the findings of this research segment underscore the pressing need to address the crucial issue of the limited genetic diversity in contemporary tigers, a situation that exacerbates all existing challenges, particularly in small populations where genetic drift is pronounced^71^.

### Spatial genetic variation

Determining population genetic structure can reveal the number of subpopulations within a single population and the degree of genetic isolation of subpopulations, and therefore, also be an indicator of dispersal status and mating of a given species^72^. A previous study reported that the Amur tiger in Laoyeling landscape and Southwest Primorye should belong to a single population due to geographical proximity and expected weak genetic differentiation in this population^62^. However, the genetic structure of this population had not been investigated in depth, resulting in its genetic background remaining unclear. Here, we clarify the genetic relationships within this small population, which is isolated from the large population in the Sikhote-Alin Mountains, and put forward that there might exist genetic differentiation in the cross-border region, supported by admixture, phylogenetic trees, and DAPC. Our study area is restricted to 21,254 km^2^, which contains the most suitable habitat for the Amur tiger in this region. The species selected in our research is the largest feline and has a high dispersal capacity, with the longest dispersal distance recorded in the literature being up to 270 km. Thus, the admixture of the entire population seems plausible^73^. Our study sites are geographically contiguous habitats but are within two countries; we infer that there may be some landscape factors that restrict gene flow, resulting in the current state of low genetic similarity within this “single population”.

### The low number of breeding individuals

The effective population size metric transforms the censused population into an idealized population that would be subject to genetic drift at the same rate as the observed population; it can indicate the number of breeding individuals and is generally less than the census population size^74–76^. Our estimate of the effective population size of wild Amur tigers in the study area is 7.6, obviously lower than the estimate for the Sikhote-Alin population estimate of 26 by the same method, and the summary statistics based on the approximate Bayesian computing framework, which yielded 14, while substantially below the rule of at least* N*_*e*_ = 100 that Frankham et al*.* had advocated in 2014 to avoid or minimize inbreeding depression in a wild population over the short term^32,66,77^. Unsurprisingly, the same situation was also observed in the ratio of effective population size to actual population size.

### Departure from random mating

Our results demonstrated a pessimistic situation that nearly one-third of Amur tiger individuals have either a high or medium inbreeding coefficient in this isolated population, including four high inbreeding individuals, posing a more severe inbreeding predicament than that observed in African lions and grey wolves^78,79^. The mean kinship coefficient among individuals in this small and isolated population was 0.0868, which was higher than that of the bighorn sheep (*Ovis canadensis*)^80^. Low effective population size may contribute to inbreeding, as the limited mate selection space promotes mating between related individuals, which leads to the accumulation of deleterious genes in the offspring and ultimately reduces fitness^81^. As in previous research on the Guam kingfisher (*Todiramphus cinnamominus*), for example, the deleterious trait of inbreeding depression may be the main reason for the rapid population decline^82^. Therefore, future studies need to keep an eye on the current status of inbreeding in this population, as well as the implementation of measures to alleviate inbreeding pressure.

### The future viability of the population

In the recent IUCN Red List assessment, the number of individuals in Russia ranged from 265 to 486, with stable population trends due to no significant changes in the three region-wide surveys since 2000. In China, 55 individuals were recorded through camera trap surveys, and multiple surveys suggest a growing population in the Laoyeling region^36^. All these results give us a promising sign of the recovery of the Amur tiger population. Nevertheless, our Vortex analysis results simulated the future population dynamics of the wild Amur tiger population across the Sino-Russian border without ongoing management actions and indicated that this isolated population is at a tremendous risk of extinction if the lethal equivalent value reaches 6.26, which challenges the optimism about the future persistence of the big cat. The probability of extinction for this population was notably elevated compared to that of the Amur leopard population with identical lethal equivalent value, and it also surpasses that of the jaguar (*Panthera onca*) with the same population size^59,83^. Therefore, we simulated rescue measures aimed at increasing the number of female individuals or reducing the proportion of harmful allele genes in order to find more effective ways to reduce the risk of population extinction. The simulation results suggest that the population may not face extinction when reproductive female individuals reach 75%, but reducing the ratio of lethal alleles proves challenging in decline (Fig. 4). Thus, in line with our third hypothesis, we believe that the optimal solution to rescue this population is to release suitable breeding female Amur tiger individuals. Therefore, the subsequent course of action for the scientific and practical aspects of tiger conservation in Northeast Asia should thoroughly consider the possibility of augmenting the population of breeding female individuals as a means to restore this charismatic species. It is inevitable that the results of our study are limited by the use of only one type of genetic marker, i.e., Microsatellite (STRs). Future studies will incorporate additional genetic data to strengthen the conclusions.

### Supplementary Information

Supplementary Information 1.

Supplementary Information 2.

Supplementary Information 3.

Supplementary Information 4.

Supplementary Information 5.

Supplementary Information 6.

Supplementary Information 7.

Supplementary Information 8.

Supplementary Information 9.

Supplementary Information 10.

Supplementary Information 11.

Supplementary Information 12.

## Data Availability

The data that support the findings of this study are openly available in raw data format in the supplementary material.
