# Supplementary figures and images for: The genetic status and rescue measure for a geographically isolated population of Amur tigers
Source: Sci Rep. 2024 Apr 6;14:8088. doi: 10.1038/s41598-024-58746-9 (PMC10998829; doi:10.1038/s41598-024-58746-9)

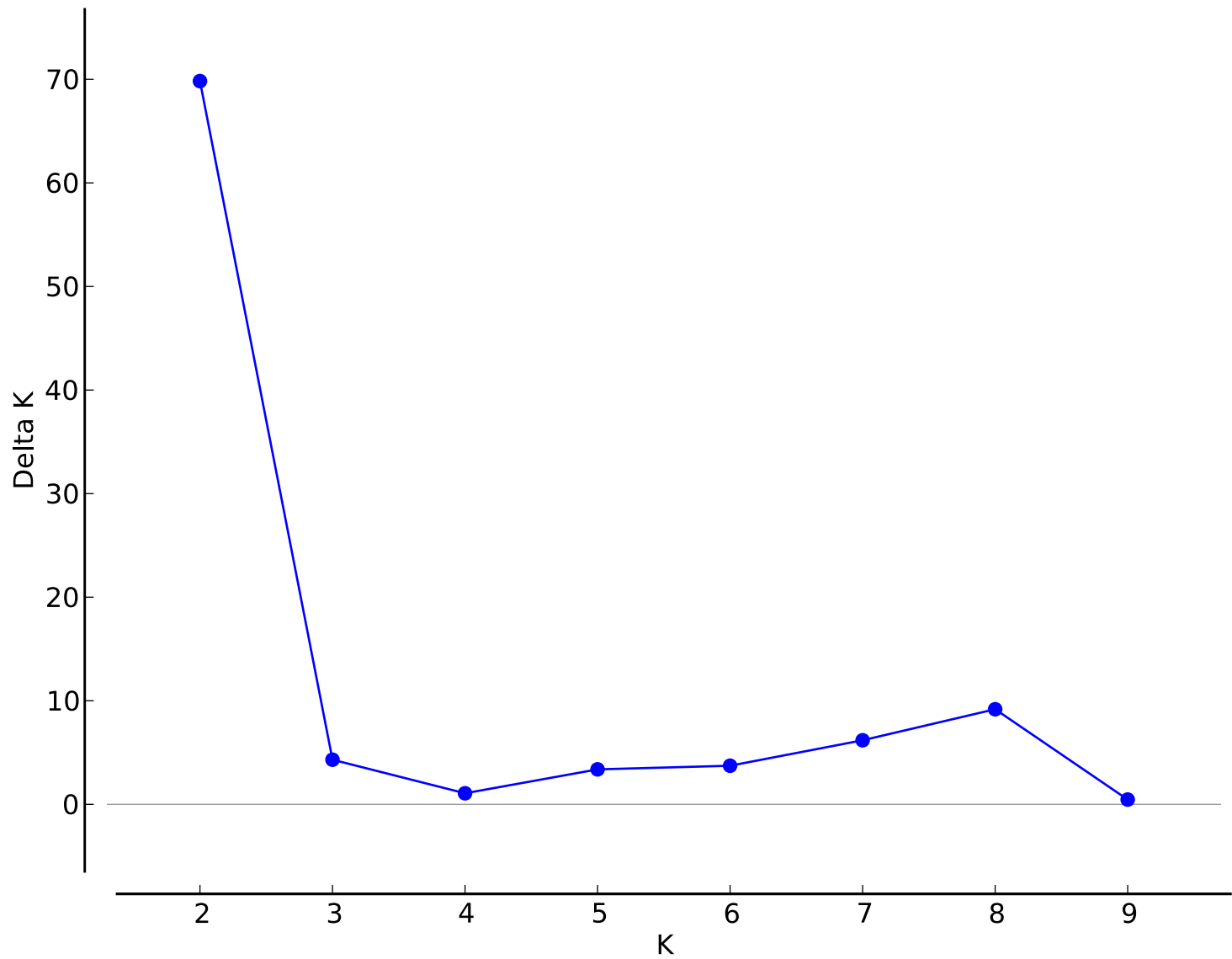

Supplement: Supplementary file 1 — Supplementary Information 1. [file 41598_2024_58746_MOESM1_ESM.pdf]

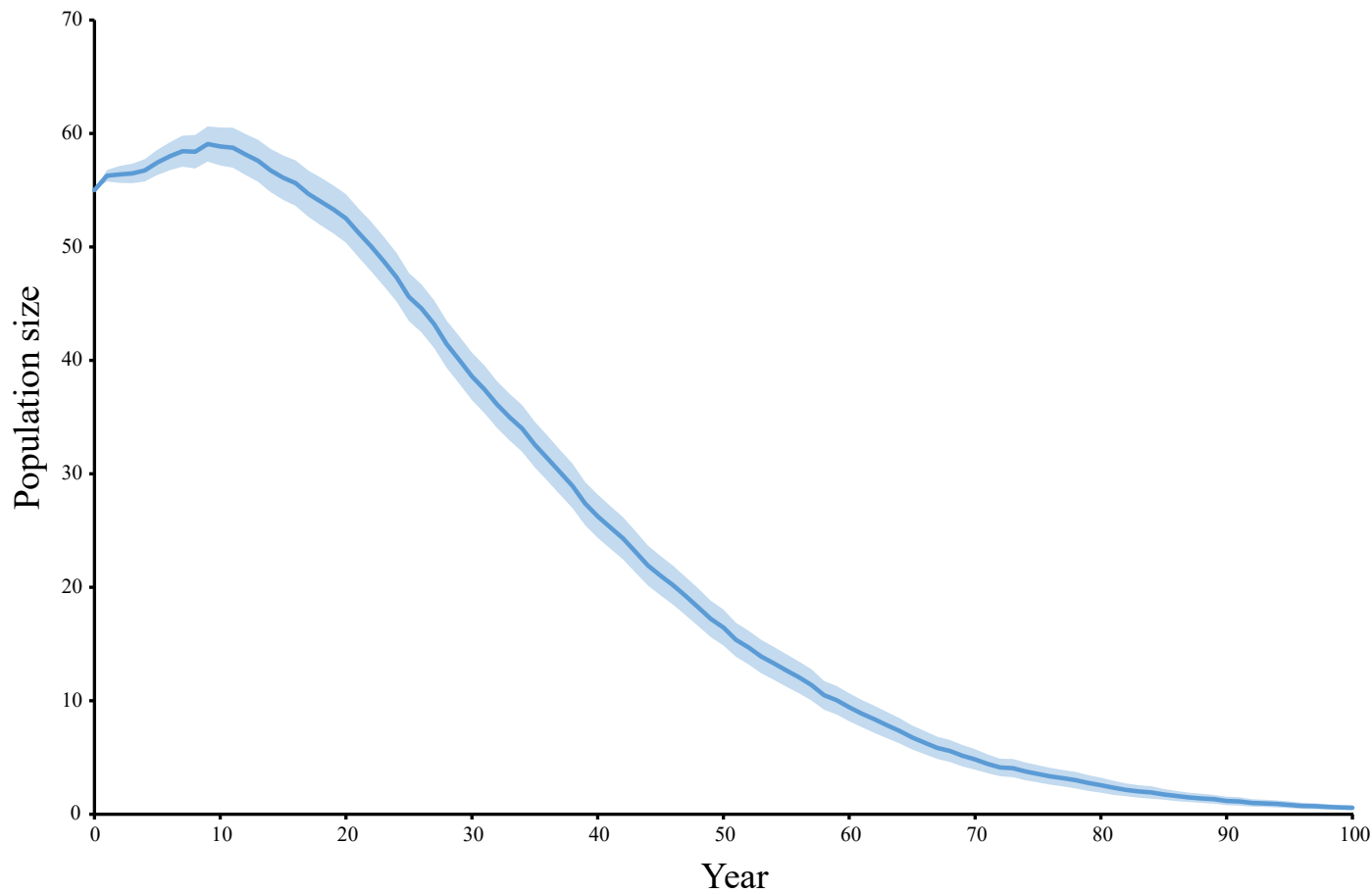

Supplement: Supplementary file 2 — Supplementary Information 2. [file 41598_2024_58746_MOESM2_ESM.pdf]
