# Supplementary material for: The genetic status and rescue measure for a geographically isolated population of Amur tigers
Source: Sci Rep. 2024 Apr 6;14:8088. doi: 10.1038/s41598-024-58746-9 (PMC10998829; doi:10.1038/s41598-024-58746-9)
Supplement: Supplementary file 4 — Supplementary Information 4. [file 41598_2024_58746_MOESM4_ESM.docx]

| Input variable | Value | Description |
| --- | --- | --- |
| Number of (iterations/year) | 1000/100 |  |
| Duration of each year | 365 |  |
| Extinction defined as | One sex |  |
| Number of populations | 1 |  |
| Lethal equivalents | 6.26 | Harihar A *et al.* (2018)^1^ |
| Percent due to recessive lethal  EV correlation between | 50 | Default |
| Reproduction and survival | 0.5 | Default |
| Polygynous |  | Empirical |
| Age of first offspring (females/males) | 3/4 | Kerley LL *et al.* (2003)^2^  Harihar A *et al.* (2018)^1^ |
| Maximum age of (female/male) reproduction | 12/12 | Harihar A *et al.* (2018)^1^ |
| Maximum lifespan | 15 | Shevtsova E *et al.* (2018)^3^ |
| Maximum number of (broods/progeny) per year | 1/6 | Empirical;* |
| Sex ratio at birth-in males | 49.5% | * |
| Offspring dependent on their mother | 2 years | Shevtsova E *et al.* (2018)^3^ |
| % adult females breeding | 50 | Harihar A *et al.* (2018)^1^ |
| SD in % breeding due to EV  distribution of broods per year | 10 | Harihar A *et al.* (2018)^1^ |
| 0 broods/1 broods | 32/68 | * |
| Specify the percent of (1/2/3/4/5/6) offspring | 5.018/19.176/28.959/ 35.168/7.326/4.353 | * |
| Mortality from age (0-1/1-2/2-3/over 3) for female | 40/10/30/10 | Harihar A *et al.* (2018)^1^ |
| SD in (0-1/1-2/2-3/over 3) mortality due to EV | 10/5/5/5 |  |
| Mortality from age (0-1/1-2/2-3/3-4/over 4) for male | 40/10/30/35/20 |  |
| SD in (0-1/1-2/2-3/3-4/over 4) mortality due to EV | 10/5/5/5/5 |  |
| % males in breeding pool | 50 | Harihar A *et al.* (2018)^1^ |
| Use age distribution | Stable | Harihar A e*t al.* (2018)^1^ |
| Initial population size | 55 | Shevtsova E *et al.* (2018)^3^ |
| Carrying capacity | 164 | See method  Empirical |
| SD in K due to EV | 5 |  |
| Number of neutral nuclear loci to be mutation  Loci to be included in summary statistics | 14  Additional loci only |  |
| Number of loci to be subject to mutation | 14 / 0.0001 | Jackson HA *et al.* (2022)^4^ |
| Start population with all inbreeding and kinship | 0.0868 | See result |

Table S1 Summary of input parameters for population viability analysis in Vortex software and the rationale behind parameter value selections.

* Last 5 years of basic data obtained from a tiger park with more than 400 captive Amur tigers.

1. Harihar, A., et al. Recovery planning towards doubling wild tiger Panthera tigris numbers: Detailing 18 recovery sites from across the range. *PLoS One* **13**, e0207114 (2018).

2. Kerley, L. L., Goodrich J. M., Miquelle D. G., Smirnov E. N., Quigley H. B. & Hornocker M. G. Reproductive parameters of wild female Amur (Siberian) tigers (Panthera tigris altaica). *Journal of Mammalogy* **84**, 288-298 (2003).

3. Shevtsova, E., Jiang G., Vitkalova A., Gu J., Qi J. & Chaika M. Saving the Amur Tiger and Amur Leopard. *Incheon: NEASPEC*, (2018).

4. Jackson, H. A., et al. Genomic erosion in a demographically recovered bird species during conservation rescue. *Conservation Biology* **36**, e13918 (2022).

**References**
