# Supplementary material for: The genetic status and rescue measure for a geographically isolated population of Amur tigers
Source: Sci Rep. 2024 Apr 6;14:8088. doi: 10.1038/s41598-024-58746-9 (PMC10998829; doi:10.1038/s41598-024-58746-9)
Supplement: Supplementary file 5 — Supplementary Information 5. [file 41598_2024_58746_MOESM5_ESM.docx]

| Table S2 Detailed information on the number of suspected Amur tiger fecal samples collected during different seasons of each year from 2013 to 2023 in China and Russia. | | | | | | | | | | | |
| --- | --- | --- | --- | --- | --- | --- | --- | --- | --- | --- | --- |
| Country | Sampling periods | 2013 | 2014 | 2015 | 2016 | 2017 | 2018 | 2019 | 2021 | 2022 | 2023 |
| China | Spring | 9 | 5 | 0 | 1 | 0 | 0 | 0 | 13 | 2 | 25 |
|  | Winter | 6 | 16 | 13 | 7 | 26 | 5 | 3 | 0 | 1 | 0 |
| Russia | Winter | 0 | 0 | 78 | 0 | 0 | 0 | 0 | 0 | 0 | 0 |
