# Supplementary material for: The genetic status and rescue measure for a geographically isolated population of Amur tigers
Source: Sci Rep. 2024 Apr 6;14:8088. doi: 10.1038/s41598-024-58746-9 (PMC10998829; doi:10.1038/s41598-024-58746-9)
Supplement: Supplementary file 6 — Supplementary Information 6. [file 41598_2024_58746_MOESM6_ESM.docx]

Table S3 Hardy-Weinberg equilibrium test was conducted using the Markov chain method, and the global estimate of Fis values was calculated according to Robertson and Hill (1984) (R&H) method.

| locus | Hardy-Weinberg | Fis estimates |
| --- | --- | --- |
| FCA32 | 0.0052 | 0.1616 |
| FCA43 | 0.0935 | 0.1112 |
| FCA44 | 0.7774 | -0.0498 |
| FCA69 | 0.1549 | 0.3376 |
| FCA90 | 1 | -0.0597 |
| FCA94 | 0.0689 | 0.3915 |
| FCA105 | 0.952 | -0.0682 |
| FCA161 | 0.9697 | -0.0283 |
| FCA176 | 0.5866 | -0.1937 |
| FCA220 | **0.0002*** | 0.0239 |
| FCA290 | 0.357 | -0.0091 |
| FCA293 | 1 | -0.1028 |
| FCA304 | 0.0876 | -0.0802 |
| FCA310 | **0.0022*** | 0.2799 |

Note: ***** represent the locus that significantly deviate from Hardy-Weinberg equilibrium.
