# Supplementary material for: The genetic status and rescue measure for a geographically isolated population of Amur tigers
Source: Sci Rep. 2024 Apr 6;14:8088. doi: 10.1038/s41598-024-58746-9 (PMC10998829; doi:10.1038/s41598-024-58746-9)
Supplement: Supplementary file 7 — Supplementary Information 7. [file 41598_2024_58746_MOESM7_ESM.docx]

Table S4 *P*-values for linkage disequilibrium were obtained using the Exact test with a dataset consisting of 14 microsatellite loci and 30 individuals as a whole.

| Pop | Locus#1 | Locus#2 | P-Value | S.E. |
| --- | --- | --- | --- | --- |
| Global | FCA32 | FCA43 | 0.62252 | 0.019789 |
| Global | FCA32 | FCA44 | 0.58754 | 0.016529 |
| Global | FCA43 | FCA44 | 0.17676 | 0.016738 |
| Global | FCA32 | FCA69 | 0.03257 | 0.005692 |
| Global | FCA43 | FCA69 | 0.27579 | 0.021034 |
| Global | FCA44 | FCA69 | 0.08503 | 0.012414 |
| Global | FCA32 | FCA90 | 0.9678 | 0.001861 |
| Global | FCA43 | FCA90 | 0.08791 | 0.006456 |
| Global | FCA44 | FCA90 | 0.00482 | 0.000847 |
| Global | FCA69 | FCA90 | 0.34495 | 0.011447 |
| Global | FCA32 | FCA94 | 0.10403 | 0.011607 |
| Global | FCA43 | FCA94 | 0.19785 | 0.018176 |
| Global | FCA44 | FCA94 | 0.06994 | 0.007527 |
| Global | FCA69 | FCA94 | 0.13641 | 0.015122 |
| Global | FCA90 | FCA94 | 0.28543 | 0.005189 |
| Global | FCA32 | FCA105 | 0.09784 | 0.004834 |
| Global | FCA43 | FCA105 | 0.83585 | 0.010171 |
| Global | FCA44 | FCA105 | 0.02109 | 0.003288 |
| Global | FCA69 | FCA105 | 0.4432 | 0.012138 |
| Global | FCA90 | FCA105 | 0.59317 | 0.006237 |
| Global | FCA94 | FCA105 | 0.08601 | 0.006445 |
| Global | FCA32 | FCA161 | 0.00595 | 0.001922 |
| Global | FCA43 | FCA161 | 0.07874 | 0.011543 |
| Global | FCA44 | FCA161 | 0.40917 | 0.017812 |
| Global | FCA69 | FCA161 | 0.48681 | 0.024417 |
| Global | FCA90 | FCA161 | 0.71518 | 0.010415 |
| Global | FCA94 | FCA161 | 0.1586 | 0.016302 |
| Global | FCA105 | FCA161 | 0.55701 | 0.019225 |
| Global | FCA32 | FCA176 | 0.98508 | 0.001289 |
| Global | FCA43 | FCA176 | 0.29622 | 0.0163 |
| Global | FCA44 | FCA176 | 0.58353 | 0.013465 |
| Global | FCA69 | FCA176 | 0.72983 | 0.016688 |
| Global | FCA90 | FCA176 | 0.92024 | 0.00246 |
| Global | FCA94 | FCA176 | 0.29697 | 0.009352 |
| Global | FCA105 | FCA176 | 0.5348 | 0.010005 |
| Global | FCA161 | FCA176 | 0.08029 | 0.006242 |
| Global | FCA32 | FCA220 | 0.02157 | 0.003854 |
| Global | FCA43 | FCA220 | 0.0358 | 0.008296 |
| Global | FCA44 | FCA220 | 0.01436 | 0.00297 |
| Global | FCA69 | FCA220 | 0.0001 | 0.00009 |
| Global | FCA90 | FCA220 | 0.00163 | 0.000615 |
| Global | FCA94 | FCA220 | 0.02074 | 0.002909 |
| Global | FCA105 | FCA220 | 0.41409 | 0.009701 |
| Global | FCA161 | FCA220 | 0.1251 | 0.013667 |
| Global | FCA176 | FCA220 | 0.77345 | 0.009478 |
| Global | FCA32 | FCA290 | 0.32166 | 0.022961 |
| Global | FCA43 | FCA290 | 0.05653 | 0.010439 |
| Global | FCA44 | FCA290 | 0.02987 | 0.005928 |
| Global | FCA69 | FCA290 | 0.01373 | 0.004172 |
| Global | FCA90 | FCA290 | 0.0143 | 0.002078 |
| Global | FCA94 | FCA290 | 0.00416 | 0.00196 |
| Global | FCA105 | FCA290 | 0.16438 | 0.007947 |
| Global | FCA161 | FCA290 | 0.40699 | 0.032602 |
| Global | FCA176 | FCA290 | 0.64206 | 0.014721 |
| Global | FCA220 | FCA290 | 0 | 0 |
| Global | FCA32 | FCA293 | 0.54235 | 0.008677 |
| Global | FCA43 | FCA293 | 0.11367 | 0.005708 |
| Global | FCA44 | FCA293 | 0.00086 | 0.000483 |
| Global | FCA69 | FCA293 | 0.13364 | 0.004925 |
| Global | FCA90 | FCA293 | 0 | 0 |
| Global | FCA94 | FCA293 | 0.09666 | 0.004835 |
| Global | FCA105 | FCA293 | 0.66115 | 0.006157 |
| Global | FCA161 | FCA293 | 0.22597 | 0.008693 |
| Global | FCA176 | FCA293 | 0.78628 | 0.005548 |
| Global | FCA220 | FCA293 | 0 | 0 |
| Global | FCA290 | FCA293 | 0.00034 | 0.000147 |
| Global | FCA32 | FCA304 | 0.37918 | 0.010781 |
| Global | FCA43 | FCA304 | 0.87806 | 0.01086 |
| Global | FCA44 | FCA304 | 0.2776 | 0.013396 |
| Global | FCA69 | FCA304 | 0.00571 | 0.001932 |
| Global | FCA90 | FCA304 | 0.76792 | 0.006573 |
| Global | FCA94 | FCA304 | 0.0338 | 0.005179 |
| Global | FCA105 | FCA304 | 0.34528 | 0.009324 |
| Global | FCA161 | FCA304 | 0.1644 | 0.017503 |
| Global | FCA176 | FCA304 | 0.67955 | 0.015831 |
| Global | FCA220 | FCA304 | 0.13157 | 0.012058 |
| Global | FCA290 | FCA304 | 0.01218 | 0.005029 |
| Global | FCA293 | FCA304 | 0.49519 | 0.007557 |
| Global | FCA32 | FCA310 | 0.10137 | 0.006699 |
| Global | FCA43 | FCA310 | 0.08969 | 0.008609 |
| Global | FCA44 | FCA310 | 0.28536 | 0.013552 |
| Global | FCA69 | FCA310 | 0.29047 | 0.011108 |
| Global | FCA90 | FCA310 | 0.01138 | 0.001271 |
| Global | FCA94 | FCA310 | 0.12585 | 0.00729 |
| Global | FCA105 | FCA310 | 0.34367 | 0.006765 |
| Global | FCA161 | FCA310 | 0.5469 | 0.013539 |
| Global | FCA176 | FCA310 | 0.96719 | 0.002586 |
| Global | FCA220 | FCA310 | 0.05977 | 0.004394 |
| Global | FCA290 | FCA310 | 0.73527 | 0.017481 |
| Global | FCA293 | FCA310 | 0.00299 | 0.000502 |
| Global | FCA304 | FCA310 | 0.26438 | 0.013212 |
