# Supplementary material for: The genetic status and rescue measure for a geographically isolated population of Amur tigers
Source: Sci Rep. 2024 Apr 6;14:8088. doi: 10.1038/s41598-024-58746-9 (PMC10998829; doi:10.1038/s41598-024-58746-9)
Supplement: Supplementary file 8 — Supplementary Information 8. [file 41598_2024_58746_MOESM8_ESM.docx]

Table S5 Summary statistics for 14 polymorphic microsatellites (*N*=30). The number of alleles (*N_a_*), observed heterozygosity (*H*_o_), expected heterozygosity (*H*_E_), and Polymorphic Information Content (*PIC*) were identified.

| *Locus* | *N_a_* | *H_o_* | *H_E_* | *PIC* |
| --- | --- | --- | --- | --- |
| *FCA32* | *4* | *0.286* | *0.502* | *0.415* |
| *FCA43* | *5* | *0.759* | *0.708* | *0.638* |
| *FCA44* | *5* | *0.667* | *0.582* | *0.535* |
| *FCA69* | *4* | *0.483* | *0.621* | *0.556* |
| *FCA90* | *2* | *0.500* | *0.473* | *0.356* |
| *FCA94* | *4* | *0.448* | *0.570* | *0.477* |
| *FCA105* | *3* | *0.633* | *0.615* | *0.531* |
| *FCA161* | *5* | *0.759* | *0.743* | *0.681* |
| *FCA176* | *3* | *0.731* | *0.595* | *0.501* |
| *FCA220* | *4* | *0.767* | *0.562* | *0.471* |
| *FCA290* | *5* | *0.741* | *0.755* | *0.699* |
| *FCA293* | *2* | *0.400* | *0.364* | *0.294* |
| *FCA304* | *3* | *0.733* | *0.632* | *0.547* |
| *FCA310* | *3* | *0.500* | *0.654* | *0.568* |
| *Mean* | *3.714* | *0.600* | *0.598* | *0.519* |
