# Supplementary material for: The genetic status and rescue measure for a geographically isolated population of Amur tigers
Source: Sci Rep. 2024 Apr 6;14:8088. doi: 10.1038/s41598-024-58746-9 (PMC10998829; doi:10.1038/s41598-024-58746-9)
Supplement: Supplementary file 9 — Supplementary Information 9. [file 41598_2024_58746_MOESM9_ESM.docx]

| Individual number | Q value | |
| --- | --- | --- |
| IND.01 | 0.967 | 0.033 |
| IND.02 | 0.956 | 0.044 |
| IND.03 | 0.946 | 0.054 |
| IND.04 | 0.934 | 0.066 |
| IND.05 | 0.934 | 0.066 |
| IND.06 | 0.933 | 0.067 |
| IND.07 | 0.918 | 0.082 |
| IND.08 | 0.91 | 0.09 |
| IND.09 | 0.896 | 0.104 |
| IND.10 | 0.888 | 0.112 |
| IND.11 | 0.855 | 0.145 |
| IND.12 | 0.83 | 0.17 |
| IND.13 | 0.809 | 0.191 |
| IND.14 | 0.808 | 0.192 |
| IND.15 | 0.747 | 0.253 |
| IND.16 | 0.744 | 0.256 |
| IND.17 | 0.709 | 0.291 |
| IND.18 | 0.558 | 0.442 |
| IND.19 | 0.47 | 0.53 |
| IND.20 | 0.289 | 0.711 |
| IND.21 | 0.166 | 0.834 |
| IND.22 | 0.137 | 0.863 |
| IND.23 | 0.1 | 0.9 |
| IND.24 | 0.084 | 0.916 |
| IND.25 | 0.069 | 0.931 |
| IND.26 | 0.059 | 0.941 |
| IND.27 | 0.051 | 0.949 |
| IND.28 | 0.042 | 0.958 |
| IND.29 | 0.035 | 0.965 |
| IND.30 | 0.033 | 0.967 |
|  |  |  |

Table S6 The overall membership proportion of two clusters were determined by using STRUCTURE.
