# Supplementary material for: The genetic status and rescue measure for a geographically isolated population of Amur tigers
Source: Sci Rep. 2024 Apr 6;14:8088. doi: 10.1038/s41598-024-58746-9 (PMC10998829; doi:10.1038/s41598-024-58746-9)
Supplement: Supplementary file 10 — Supplementary Information 10. [file 41598_2024_58746_MOESM10_ESM.docx]

Table S7 Genetic distances of *Dsw* were calculated for a cohort of 30 individuals.

|  | IND.01 | IND.02 | IND.03 | IND.04 | IND.05 | IND.06 | IND.07 | IND.08 | IND.09 | IND.10 | IND.11 | IND.13 | IND.14 | IND.15 | IND.17 | IND.20 | IND.21 | IND.23 | IND.24 | IND.25 | IND.30 | IND.12 | IND.16 | IND.18 | IND.19 | IND.22 | IND.26 | IND.27 | IND.28 |
| --- | --- | --- | --- | --- | --- | --- | --- | --- | --- | --- | --- | --- | --- | --- | --- | --- | --- | --- | --- | --- | --- | --- | --- | --- | --- | --- | --- | --- | --- |
| IND.01 | 0 |  |  |  |  |  |  |  |  |  |  |  |  |  |  |  |  |  |  |  |  |  |  |  |  |  |  |  |  |
| IND.02 | 0.250 |  |  |  |  |  |  |  |  |  |  |  |  |  |  |  |  |  |  |  |  |  |  |  |  |  |  |  |  |
| IND.03 | 0.500 | 0.125 |  |  |  |  |  |  |  |  |  |  |  |  |  |  |  |  |  |  |  |  |  |  |  |  |  |  |  |
| IND.04 | 0.404 | 0.196 | 0.250 |  |  |  |  |  |  |  |  |  |  |  |  |  |  |  |  |  |  |  |  |  |  |  |  |  |  |
| IND.05 | 0.442 | 0.286 | 0.518 | 0.482 |  |  |  |  |  |  |  |  |  |  |  |  |  |  |  |  |  |  |  |  |  |  |  |  |  |
| IND.06 | 0.455 | 0.333 | 0.438 | 0.479 | 0.917 |  |  |  |  |  |  |  |  |  |  |  |  |  |  |  |  |  |  |  |  |  |  |  |  |
| IND.07 | 0.538 | 0.125 | 0.214 | 0.179 | 0.304 | 0.646 |  |  |  |  |  |  |  |  |  |  |  |  |  |  |  |  |  |  |  |  |  |  |  |
| IND.08 | 0.269 | 0.173 | 0.231 | 0.135 | 0.442 | 0.432 | 0.231 |  |  |  |  |  |  |  |  |  |  |  |  |  |  |  |  |  |  |  |  |  |  |
| IND.09 | 0.212 | 0.268 | 0.464 | 0.429 | 0.196 | 0.708 | 0.321 | 0.327 |  |  |  |  |  |  |  |  |  |  |  |  |  |  |  |  |  |  |  |  |  |
| IND.10 | 0.231 | 0.232 | 0.464 | 0.393 | 0.232 | 0.563 | 0.393 | 0.308 | 0.214 |  |  |  |  |  |  |  |  |  |  |  |  |  |  |  |  |  |  |  |  |
| IND.11 | 0.250 | 0.192 | 0.365 | 0.308 | 0.308 | 0.614 | 0.250 | 0.250 | 0.154 | 0.173 |  |  |  |  |  |  |  |  |  |  |  |  |  |  |  |  |  |  |  |
| IND.13 | 0.350 | 0.159 | 0.250 | 0.250 | 0.432 | 0.325 | 0.341 | 0.250 | 0.364 | 0.455 | 0.375 |  |  |  |  |  |  |  |  |  |  |  |  |  |  |  |  |  |  |
| IND.14 | 0.596 | 0.250 | 0.411 | 0.446 | 0.214 | 0.521 | 0.304 | 0.481 | 0.411 | 0.304 | 0.346 | 0.455 |  |  |  |  |  |  |  |  |  |  |  |  |  |  |  |  |  |
| IND.15 | 0.558 | 0.304 | 0.571 | 0.571 | 0.196 | 0.813 | 0.357 | 0.596 | 0.321 | 0.393 | 0.385 | 0.295 | 0.232 |  |  |  |  |  |  |  |  |  |  |  |  |  |  |  |  |
| IND.17 | 0.519 | 0.286 | 0.268 | 0.268 | 0.643 | 0.396 | 0.339 | 0.288 | 0.589 | 0.554 | 0.385 | 0.409 | 0.500 | 0.768 |  |  |  |  |  |  |  |  |  |  |  |  |  |  |  |
| IND.20 | 0.500 | 0.308 | 0.423 | 0.346 | 0.365 | 0.523 | 0.308 | 0.313 | 0.308 | 0.231 | 0.292 | 0.325 | 0.269 | 0.385 | 0.519 |  |  |  |  |  |  |  |  |  |  |  |  |  |  |
| IND.21 | 0.538 | 0.519 | 0.769 | 0.519 | 0.596 | 0.818 | 0.615 | 0.577 | 0.481 | 0.385 | 0.442 | 0.475 | 0.558 | 0.596 | 0.865 | 0.333 |  |  |  |  |  |  |  |  |  |  |  |  |  |
| IND.23 | 0.558 | 0.357 | 0.375 | 0.446 | 0.286 | 0.875 | 0.339 | 0.365 | 0.375 | 0.446 | 0.385 | 0.455 | 0.393 | 0.446 | 0.429 | 0.327 | 0.712 |  |  |  |  |  |  |  |  |  |  |  |  |
| IND.24 | 0.769 | 0.464 | 0.625 | 0.732 | 0.393 | 0.729 | 0.554 | 0.654 | 0.661 | 0.589 | 0.596 | 0.636 | 0.321 | 0.446 | 0.500 | 0.519 | 0.923 | 0.321 |  |  |  |  |  |  |  |  |  |  |  |
| IND.25 | 0.712 | 0.393 | 0.518 | 0.696 | 0.429 | 0.792 | 0.446 | 0.635 | 0.554 | 0.589 | 0.538 | 0.523 | 0.393 | 0.446 | 0.464 | 0.442 | 0.942 | 0.250 | 0.107 |  |  |  |  |  |  |  |  |  |  |
| IND.30 | 0.788 | 0.536 | 0.696 | 0.696 | 0.536 | 1.021 | 0.482 | 0.827 | 0.411 | 0.589 | 0.423 | 0.591 | 0.464 | 0.411 | 0.857 | 0.423 | 0.596 | 0.500 | 0.679 | 0.536 |  |  |  |  |  |  |  |  |  |
| IND.12 | 0.519 | 0.321 | 0.589 | 0.482 | 0.250 | 0.896 | 0.304 | 0.481 | 0.411 | 0.411 | 0.346 | 0.523 | 0.357 | 0.339 | 0.571 | 0.462 | 0.712 | 0.393 | 0.500 | 0.429 | 0.643 |  |  |  |  |  |  |  |  |
| IND.16 | 0.654 | 0.339 | 0.536 | 0.536 | 0.268 | 0.958 | 0.286 | 0.538 | 0.321 | 0.321 | 0.173 | 0.659 | 0.268 | 0.357 | 0.625 | 0.385 | 0.654 | 0.482 | 0.589 | 0.518 | 0.411 | 0.268 |  |  |  |  |  |  |  |
| IND.18 | 0.615 | 0.321 | 0.268 | 0.339 | 0.679 | 0.479 | 0.339 | 0.385 | 0.625 | 0.625 | 0.404 | 0.455 | 0.500 | 0.768 | 0.071 | 0.596 | 0.923 | 0.464 | 0.536 | 0.464 | 0.786 | 0.536 | 0.554 |  |  |  |  |  |  |
| IND.19 | 0.538 | 0.286 | 0.446 | 0.339 | 0.286 | 0.688 | 0.196 | 0.385 | 0.375 | 0.411 | 0.212 | 0.568 | 0.250 | 0.411 | 0.286 | 0.404 | 0.615 | 0.321 | 0.357 | 0.357 | 0.464 | 0.250 | 0.232 | 0.250 |  |  |  |  |  |
| IND.22 | 0.550 | 0.455 | 0.477 | 0.795 | 0.477 | 0.944 | 0.568 | 0.700 | 0.523 | 0.500 | 0.575 | 0.781 | 0.659 | 0.682 | 0.682 | 0.591 | 0.875 | 0.318 | 0.523 | 0.341 | 0.795 | 0.386 | 0.500 | 0.636 | 0.500 |  |  |  |  |
| IND.26 | 0.885 | 0.554 | 0.750 | 0.750 | 0.518 | 0.750 | 0.536 | 0.731 | 0.679 | 0.714 | 0.635 | 0.705 | 0.375 | 0.464 | 0.554 | 0.558 | 0.962 | 0.482 | 0.268 | 0.304 | 0.661 | 0.375 | 0.500 | 0.446 | 0.304 | 0.727 |  |  |  |
| IND.27 | 0.977 | 0.646 | 0.771 | 0.958 | 0.458 | 0.864 | 0.625 | 0.886 | 0.604 | 0.750 | 0.659 | 0.700 | 0.438 | 0.354 | 0.750 | 0.614 | 1.023 | 0.500 | 0.313 | 0.292 | 0.625 | 0.396 | 0.521 | 0.625 | 0.438 | 0.775 | 0.063 |  |  |
| IND.28 | 0.667 | 0.423 | 0.462 | 0.635 | 0.519 | 0.727 | 0.442 | 0.604 | 0.442 | 0.481 | 0.333 | 0.675 | 0.500 | 0.615 | 0.462 | 0.458 | 0.771 | 0.462 | 0.558 | 0.404 | 0.442 | 0.519 | 0.288 | 0.385 | 0.365 | 0.591 | 0.481 | 0.563 |  |
| IND.29 | 0.896 | 0.654 | 0.788 | 0.750 | 0.692 | 1.208 | 0.596 | 0.708 | 0.654 | 0.519 | 0.500 | 0.955 | 0.731 | 0.827 | 0.712 | 0.500 | 0.833 | 0.481 | 0.788 | 0.577 | 0.692 | 0.596 | 0.538 | 0.712 | 0.596 | 0.350 | 0.846 | 0.750 | 0.396 |
