# Supplementary material for: The genetic status and rescue measure for a geographically isolated population of Amur tigers
Source: Sci Rep. 2024 Apr 6;14:8088. doi: 10.1038/s41598-024-58746-9 (PMC10998829; doi:10.1038/s41598-024-58746-9)
Supplement: Supplementary file 11 — Supplementary Information 11. [file 41598_2024_58746_MOESM11_ESM.docx]

| Table S8 The values of *Ne* under different *Pcrit* were estimated using the linkage disequilibrium method. | | | | | |
| --- | --- | --- | --- | --- | --- |
| Lowest Allele Frequency Used | 0.05 | 0.02 | 0.01 | No S* | 0+ |
| Estimated Ne^ = | 7.2 | 7.6 | 8 | 7.6 | 8 |
| 95% CIs for Ne^* Parametric | 4.7 | 5.1 | 5.4 | 5.1 | 5.4 |
|  | 10.4 | 10.8 | 11.5 | 10.8 | 11.5 |
| * Jackknife on Samples | 3.2 | 3.3 | 5 | 3.2 | 5 |
|  | 12.4 | 13.6 | 12.2 | 14 | 12.2 |
