# Supplementary material for: The genetic status and rescue measure for a geographically isolated population of Amur tigers
Source: Sci Rep. 2024 Apr 6;14:8088. doi: 10.1038/s41598-024-58746-9 (PMC10998829; doi:10.1038/s41598-024-58746-9)
Supplement: Supplementary file 12 — Supplementary Information 12. [file 41598_2024_58746_MOESM12_ESM.docx]

|  | Ritland | LynchRd | TrioML | DyadML |
| --- | --- | --- | --- | --- |
| IND.01 | 0.0926 | 0.1457 | 0.1683 | 0.2026 |
| IND.02 | -0.2449 | -0.3926 | 0 | 0 |
| IND.03 | -0.0366 | -0.052 | 0.0357 | 0.0473 |
| IND.04 | 0.0469 | 0.0575 | 0.1515 | 0.1755 |
| IND.05 | -0.0432 | -0.0704 | 0.0155 | 0.0223 |
| IND.06 | 0.1883 | 0.2836 | 0.274 | 0.3073 |
| IND.07 | -0.0744 | -0.1281 | 0.0181 | 0.024 |
| IND.08 | -0.0122 | -0.0096 | 0.0584 | 0.0802 |
| IND.09 | -0.0926 | -0.1436 | 0.023 | 0.0313 |
| IND.10 | -0.0956 | -0.1251 | 0.0175 | 0.0259 |
| IND.11 | -0.2225 | -0.3903 | 0 | 0 |
| IND.12 | 0.0691 | 0.127 | 0.1181 | 0.1604 |
| IND.13 | -0.0675 | -0.0351 | 0.0285 | 0.0367 |
| IND.14 | -0.0402 | -0.0672 | 0.0259 | 0.039 |
| IND.15 | 0.4723 | 0.439 | 0.1655 | 0.2075 |
| IND.16 | -0.0846 | -0.1423 | 0.005 | 0.0079 |
| IND.17 | -0.03 | -0.0452 | 0.0293 | 0.0333 |
| IND.18 | -0.0809 | -0.0968 | 0.0097 | 0.0134 |
| IND.19 | -0.1325 | -0.19 | 0.0027 | 0.0028 |
| IND.20 | -0.2132 | -0.1908 | 0.0013 | 0.0018 |
| IND.21 | 1.1706 | 0.4061 | 0.2195 | 0.2553 |
| IND.22 | 0.3142 | 0.3602 | 0.3084 | 0.3429 |
| IND.23 | -0.2766 | -0.2819 | 0 | 0 |
| IND.24 | -0.0542 | -0.0639 | 0.0245 | 0.0361 |
| IND.25 | -0.0881 | -0.1016 | 0.0129 | 0.0177 |
| IND.26 | -0.0438 | -0.0336 | 0.0245 | 0.0317 |
| IND.27 | 0.1784 | 0.1532 | 0.2023 | 0.2262 |
| IND.28 | -0.1301 | -0.0528 | 0.0062 | 0.007 |
| IND.29 | 0.3535 | 0.2662 | 0.3013 | 0.3362 |
| IND.30 | 0.7613 | 0.4363 | 0.3563 | 0.3647 |
|  |  |  |  |  |

Table S9 The inbreeding coefficient of 30 individuals were calculated using four different methodologies.
